# Nonlinear Charge Transport and Excitable Phenomena in Semiconductor Superlattices

**DOI:** 10.3390/e26080672

**Published:** 2024-08-08

**Authors:** Luis L. Bonilla, Manuel Carretero, Emanuel Mompó

**Affiliations:** 1 Gregorio Millán Institute for Fluid Dynamics, Nanoscience and Industrial Mathematics, Universidad Carlos III de Madrid, 28911 Leganés, Spain; manuel.carretero@uc3m.es (M.C.); egmompo@icai.comillas.edu (E.M.); 2 Department of Mathematics, Universidad Carlos III de Madrid, 28911 Leganés, Spain; 3 Departamento de Matemática Aplicada, Grupo de Dinámica No Lineal, Universidad Pontificia Comillas, 28015 Madrid, Spain

**Keywords:** semiconductor superlattices, resonant quantum tunneling, quantum transport, excitable media, coherence resonance, stochastic resonance, self-sustained oscillations, chaos

## Abstract

Semiconductor superlattices are periodic nanostructures consisting of epitaxially grown quantum wells and barriers. For thick barriers, the quantum wells are weakly coupled and the main transport mechanism is a sequential resonant tunneling of electrons between wells. We review quantum transport in these materials, and the rate equations for electron densities, currents, and the self-consistent electric potential or field. Depending on superlattice configuration, doping density, temperature, voltage bias, and other parameters, superlattices behave as excitable systems, and can respond to abrupt dc bias changes by large transients involving charge density waves before arriving at a stable stationary state. For other parameters, the superlattices may have self-sustained oscillations of the current through them. These oscillations are due to repeated triggering and recycling of charge density waves, and can be periodic in time, quasiperiodic, and chaotic. Modifying the superlattice configuration, it is possible to attain robust chaos due to wave dynamics. External noise of appropriate strength can generate time-periodic current oscillations when the superlattice is in a stable stationary state without noise, which is called the coherence resonance. In turn, these oscillations can resonate with a periodic signal in the presence of sufficient noise, thereby displaying a stochastic resonance. These properties can be exploited to design and build many devices. Here, we describe detectors of weak signals by using coherence and stochastic resonance and fast generators of true random sequences useful for safe communications and storage.

## 1. Introduction

**Figure 1:**
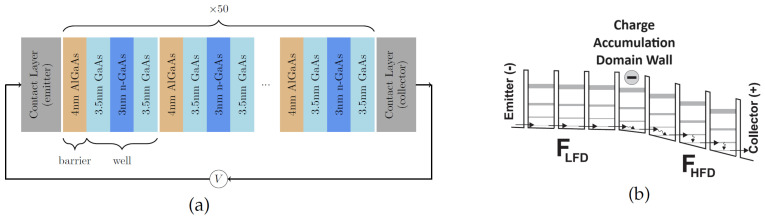
(**a**) Sketch of a voltage biased semiconductor superlattice. An epitaxially grown succession of alternate layers of two semiconductors is cut into a mesa, whose cross section is a square (or a circle) with sides measuring tens of microns. The semiconductor with smaller (larger) bandgap forms the QWs (QBs) of the superlattice conduction band. Here, QWs are 10 nm layers of GaAs negatively doped in their central part, and QBs are 4 nm undoped layers of AlGaAs. (**b**) Sketch of a stationary electric potential profile in the SSL conduction band, comprising a LFD followed by a charge accumulation domain wall and a HFD. In the LFD, sequential resonant tunneling of electrons is from the lowest subband to the lowest subband of the adjacent QW across the QB. In the HFD, electrons tunnel from lowest subband to first excited subband of the adjacent QW, followed by a fast scattering event that transfers electrons to the lowest subband of the same QW.

Semiconductor superlattices (SSLs) are periodic nanostructures consisting of epitaxially grown quantum wells and barriers [1,2,3]. A simple SSL consists of many periods, each comprising two layers of semiconductors with different energy gaps but similar lattice constants. The structure is cut into a square or circular mesa, whose lateral extension is much larger than the superlattice period. Quantum wells (QWs) and quantum barriers (QBs) in the conduction band of the SSL correspond to the semiconductor with a smaller and larger energy gap, respectively. Typically, two contacts are attached to the ends of the nanostructure and connected to a circuit, as sketched in Figure 1a. While SSLs were postulated by Esaki and Tsu [1] to observe Bloch oscillations, which required thin barriers to achieve miniband transport [3], many early experiments were carried out on SSLs with thick barriers [4], such that the barrier width is much larger than the typical electron wavelength inside the QB. For thick barriers, the quantum wells are weakly coupled and the main transport mechanism is the sequential resonant tunneling of electrons between wells [3,5,6,7]. Figure 1b depicts the electric potential profile of a stationary state comprising a low field domain (LFD) separated by a charge accumulation domain wall from a high field domain (HFD). In the LFD, electrons tunnel across QBs from the lowest energy level of a QW to the lowest energy level of the next QW. In the HFD, electrons tunnel from the lowest energy level of a QW to an excited state of the next QW, followed by a scattering event that brings them down to the lowest energy level before they tunnel through the next QB. We review the quantum transport in these materials and the rate equations for electron densities, currents, and the self-consistent electric potential or field, which are spatially discrete. Depending on the superlattice configuration, doping density, voltage bias, temperature, and other parameters, these superlattices behave as excitable systems, and can respond to abrupt changes in dc bias voltage by large transients involving charge density waves before arriving at a stable stationary state. They can also exhibit self-sustained oscillations of the current (SSOC) through the SSLs. The oscillations are due to repeated triggering and recycling of charge density waves, which can be periodic in time, quasiperiodic, and chaotic. Modifying the superlattice configuration, it is possible to attain robust chaos due to wave dynamics. External noise of appropriate strength can generate SSOC when the superlattice is in a stable stationary state without noise, which is called coherence resonance (CR). In turn, oscillations from coherence resonance can resonate themselves with a periodic signal in the presence of sufficient noise, thereby displaying a stochastic resonance (SR). In the last ten years, a novel design of AlGaAs/GaAs SSLs with 45% Al in their quantum barriers allows us to attain these nonlinear phenomena at room temperature [8], whereas they could be observed only at ultralow temperatures with the usual AlGa/GaAs superlattices [3]. These properties can be exploited to design and build many devices. Here, we describe detectors of weak signals by using coherence, stochastic resonance, and fast generators of true random sequences useful for safe communications and storage that exploit chaotic attractors.

What do SSLs have to do with excitable systems and media? An excitable dynamical system has a stable attractor, but has two ways to return to it when disturbed. For small disturbances from the attractor, it goes back rapidly, whereas the system undergoes a large excursion before returning provided the disturbance surpasses a finite threshold value. Spatially extended systems are excitable media when a stimulus of sufficient size can trigger a wave that will propagate through the medium. In excitable media, there is a refractory period before a similar disturbance can trigger another wave [9,10,11]. In physiology, excitable cells include cardiac and muscle cells, secretory cells, and most neurons [12]. The effects of spatial discreteness are important in many physical and biological systems consisting of interacting components, such as atoms, quantum wells, cells, etc. Besides weakly coupled SSLs, examples include atoms adsorbed on a periodic substrate [13], propagation of cracks in a brittle material [14,15], microscopic theories of friction between solid bodies [16], crystal growth and interface motion in crystalline materials [17], motion of dislocations [18,19,20,21,22], sliding of charge density waves [23], superconductor Josephson array junctions [24], pulse propagation in myelinated nerves [12], unzipping of DNA hairpins and modular proteins [25,26], etc. While these examples are very different, their common features are related to wave propagation and pinning of waves in spatially discrete systems [27]. In spatially discrete equations that have wave fronts or pulses as solutions, there may be intervals of a control parameter for which these waves have zero velocity and become stationary solutions. Outside the pinning intervals, the waves move. Excitability is related to a sudden change in the control parameter outside the pinning interval, which triggers an appropriate wave. For weakly coupled SSLs, the control parameter is dc voltage [3].

Weakly coupled SSLs are excitable media when there is a stable stationary state. It can be one branch of the multistable stationary states that appear for appropriate high doping densities, or it can be the stable stationary state past a saddle-node infinite period (SNIPER) bifurcation of a limit cycle. In both cases, the stable state has the field profile of a pinned wave front, which is a domain wall (DW) separating low field domains (LFDs) and high field domains (HFDs). The large excursion after a disturbance over a threshold consists of generation of an HFD bounded by charge accumulation and depletion DWs at the emitter, and motion of the existing DWs until a stable stationary state is reached. Accumulation and depletion DWs move at velocities that depend on the instantaneous value of the total current density. The latter satisfies a universal equation, which depends on the number and type of DWs moving on the SSL [5]. DW dynamics also explain the stages of SSOC in SSLs that have appropriately low doping density [3,5]. Under a controllable external noise, the excitability and oscillatory properties of SSLs can be exploited to produce coherence and stochastic resonances.

This paper also reviews properties of SSLs that behave as excitable and oscillatory media. The paper is organized as follows. Section 2 describes different approaches to quantum transport in SSLs, with particular emphasis on the microscopic sequential resonant tunneling model, which has different effective masses and voltage drops at quantum wells (QWs) and quantum barriers (QBs) [7,28]. Section 3 explains how weakly coupled SSLs with high doping density are excitable media in which large disturbances of a stationary state produce long excursions of the total current density until the SSL goes back to a stationary state. Section 4 shows that SSOCs appear for a certain interval of *dc* voltages for appropriate values of the SSL parameters. Using our detailed transport model, we describe how noise can change stable states of excitable SSLs in Section 5. In previous works, we used an averaged version of the detailed transport model to describe the same phenomena [29]. Starting from a *dc* voltage just outside of the region of SSOC (past a SNIPER bifurcation), sufficiently large external noise can produce a periodic oscillation of the current, which is called a coherence resonance (CR). Noise can also be used to produce a resonance between the CR and a weak sinusoidal external voltage signal immersed in noise, which is a stochastic resonance (SR) that could be used to detect weak signals. In Section 6, we explain how to insert two equally modified QWs in an otherwise ideal SSL with identical periods to produce robust chaotic dynamics [7,28]. This design can be achieved with currently available growth techniques, and it persists under reasonable disorder due to epitaxial growth and internal and external noise. Section 7 contains the conclusions of this work.

## 2. Quantum Transport in Semiconductor Superlattices

Different approaches to electron transport in SSLs are reviewed in [3,6]. Essentially, we have to choose states of single electron transport in a periodic potential as an appropriate basis, and derive a quantum kinetic equation, which is then analyzed to explain nonlinear phenomena such as excitability and oscillations. Ignoring electron–electron interaction and scattering, at a zero electric field, we can use extended Bloch states for electron minibands or localized Wannier states [6]. These one-dimensional (1D) states have to be multiplied by plane waves in the direction perpendicular to SSL growth (subband energies) [30].

If the QBs are thin, the applied electric fields are not overly large, and the minibands are wide, only the first miniband is populated. Then, a Boltzmann transport equation with appropriate collision terms describes the electron transport in the semiclassical limit for the resulting strongly coupled SSL. Electron–electron interaction is described by a Poisson equation for the electric potential in a Hartree mean field approximation, in which electron density is calculated from the Boltzmann equation. For the resulting system, it is possible to obtain hydrodynamic or drift–diffusion partial differential equations in the limit in which the Bloch frequency (proportional to the electric field) is of the same order as the collision frequency. For simple collision kernels of the Bhatnagar–Gross–Krook type, these equations can be derived explicitly by a Chapman–Enskog method [3]. A variety of SSOCs are solutions of these equations and explain many experiments; see the review [3]. If a Wigner transport equation is used instead of the Boltzmann equations, the same procedure yields nonlocal quantum drift–diffusion equations describing nonlinear SSOCs with small quantum effects [31]. For sufficiently large electric fields, electrons may be found in more than one miniband, and quantum resonant tunneling occurs during SSOC [32].

If QBs are thick, the appropriate one-electron basis for the resulting weakly coupled SSL comprises Wannier states, which can be approximated by subband states of isolated QWs. Sequential resonant tunneling is the main transport mechanism if the intrasubband scattering time to be much shorter than the intersubband scattering time, which is much shorter than the interwell tunneling time across QBs; see the reviews [3,5,6]. Then, the electrons are in local equilibrium within the subbands of each QW at the instantaneous values of electric field, average density, and chemical potential. The tunneling current between subbands of adjacent QWs is stationary, and it depends on the subband electron densities and the average electric field, which evolve on the much longer time scale of dielectric relaxation time [33]. These assumptions for weakly coupled SSLs have been validated by experiments [3]. The subband populations and the values of the electric field of each SSL period satisfy rate equations and spatially discrete Poisson equations [7]. Strictly speaking, only the subband with lowest energy of each QW is appreciably populated if the intersubband scattering time is much shorter than the interwell tunneling time, which is the usual case in sequential resonant tunneling [7]. Spatially discrete dynamics for weakly coupled SSLs were suggested by experiments by Esaki and Chang [4], and proposed by Suris [34] and later authors [33,35,36,37].

What is the appropriate description for intermediate cases between strongly and weakly coupled SSLs? We would need a one-electron basis that interpolates between them (typically, Wannier states multiplied by plane waves along the transverse direction) and a general quantum kinetic equation. Under a number of restrictions, it is possible to derive a quantum transport equation for the matrix-valued nonequilibrium Green function (NEGF) [6,38]. This equation yields the stationary tunneling current between Wannier states of adjacent QWs, provided the electric field is homogeneous and stationary. Other approaches using density matrices or Wannier–Stark states produce tunneling current densities that can also be obtained from NEGF in appropriate limits [6]. An important advantage of the NEGF approach is that numerical simulations thereof yield the current density and the current–voltage curve of the device, including appropriate modeling of scattering, which compares very well with experimental measurements [39]. NEGF can be calculated numerically for a variety of nanodevices including SSLs, quantum wires and quantum cascade lasers [39,40,41,42,43,44,45,46], and nanoscale MOSFET and transistors [47,48,49,50].

Thus, it could seem that the NEGF is the most general approach to obtain the balance rate equations for electron densities and electric fields. This is not so, because the NEGF produces stationary tunneling currents for different nanostructures only by assuming that the electric field is homogeneous and constant [6,44]. To derive rate equations describing space-dependent nonlinear phenomena, we need to postulate the same separation of time scales as in the case of weakly coupled SSLs [33]. Then, we have to replace the homogeneous constant electric field a posteriori by time-dependent local electric fields at each SSL period, and use the NEGF to calculate numerically a stationary current density to be inserted into a constitutive equation for the tunneling current [46]. In practice, we postulate the spatially discrete drift–diffusion equations coupled to the Poisson equation for the electric field. Then, we insert where needed the numerically calculated tunneling current density obtained by solving the special case of NEGF equations homogeneous in space under constant electric field [6,46]. To improve this theory beyond such a patchwork, one would need to derive the equations for QW electron densities and local electric fields directly from a space inhomogeneous NEGF coupled with the Poisson equation for the electric potential, possibly followed by some coarse-graining procedure to obtain the spatially discrete equations. To this day, this remains an open problem.

### 2.1. Rate Equations for Ideal Superlattices

In weakly coupled SSLs, QBs are thick and electrons tunnel from the lowest energy subband at one QW to subbands at the next one. If the subband to which they tunnel is an excited one, scattering may lower the electrons to the lowest one, from which they repeat the process. This mechanism is sequential resonant tunneling. Here, we shorten the description in our previous paper [7], where further details can be found. Following [7,33], we assume: *intrasubband scattering time* ≪*intersubband scattering time*≪*interwell tunneling time across barriers*≪*dielectric relaxation time*. The electrons are at local equilibrium within each subband with 2D electron densities $n_{i}^{\left(\nu \right)}$ related to their chemical potentials $\mu_{i}^{\left(\nu \right)}$ by [5]

(1) $$\begin{array}{ccc} & & n_{i}^{\left(\nu \right)}=\frac{m_{W}k_{B}T}{\pi \hbar^{2}}\int_{0}^{\infty }A_{C\nu }\left(\epsilon \right)\;\ln\;\left(1+e^{\left(\mu_{i}^{\left(\nu \right)}-\epsilon \right)/k_{B}T}\right)d\epsilon ,\;\nu =1,\ldots ,n, \end{array}$$

(2) $$\begin{array}{ccc} & & A_{C\nu }\left(\epsilon \right)=\frac{\gamma_{\nu }}{\pi }\;\frac{1}{{\left(\epsilon -E_{C\nu }\right)}^{2}+\gamma_{\nu }^{2}} \end{array}$$

Here, i=1,…,N, where *N* is the number of identical SSL periods, each with length $d_{B}+d_{W}$ ($d_{B}$ and $d_{W}$ are barrier and well widths, respectively). $E_{C\nu }$ is the energy of subband ν (measured from the bottom of the *i*th QW), and $m_{W}$, *T*, and $k_{B}$ are the electron effective mass at QWs, the lattice temperature, and the Boltzmann constant, respectively. The Lorentzian functions (2) have half-widths $\gamma_{\nu }=\hbar /\tau_{sc}$, where $\tau_{sc}$ is the lifetime associated with any scattering process dominant in the sample.

The wave function of an electron in miniband ν is $e^{iqx+k_{⊥}\cdot x_{⊥}}u_{q}^{\nu }\left(x\right)$ (a plane wave on the lateral directions $x_{⊥}=\left(y,z\right)$ times a Bloch state on the direction of the superlattice vertical growth; $u_{q}^{\nu }\left(x\right)$ is a periodic function of *x* with the SSL period). The energy minibands $\epsilon_{\nu }\left(q\right)$ corresponding to the Bloch states solve a 1D Kronig–Penney model [30]

(3) $$\begin{array}{ccc} & & \cos ql=\cos kd_{W}\cosh\alpha d_{B}-\frac{1}{2}\;\left(\frac{1}{\xi }-\xi \right)\;\sin kd_{W}\sinh\alpha d_{B},\\ & & k=\frac{\sqrt{2m_{W}\epsilon }}{\hbar },\;\alpha =\frac{\sqrt{2m_{B}\left(eV_{B}-\epsilon \right)}}{\hbar },\;\xi =\frac{m_{W}\alpha }{m_{B}k}=\sqrt{\frac{m_{W}}{m_{B}}\;\left(\frac{eV_{B}}{\epsilon }-1\right)}. \end{array}$$

In the limit, as $\alpha d_{B}\to \infty$, Equation (4) produces the subbands $\epsilon =E_{C\nu }$ appearing in Equation (2):

(4) $$\begin{array}{c} \cos kd_{W}-\frac{1}{2}\;\left(\frac{1}{\xi }-\xi \right)\;\sin kd_{W}=0. \end{array}$$

### 2.2. Charge Continuity and Tunneling Current

The constitutive relation between the tunneling current and electron densities can be obtained using density matrices [51], nonequilibrium Green functions [6,46], or the Transfer Hamiltonian [5,52,53]. Here, we use the latter approach [5,7]. Provided that the scattering times between higher subbands and those with lower energy are much smaller than the dielectric relaxation time, the first subband is the only that is appreciably populated, $n_{i}^{\left(\nu \right)}\approx 0$ for ν>1 and $n_{i}^{\left(1\right)}\approx n_{i}$, $\mu^{\left(1\right)}\approx \mu_{i}$. Then, the charge continuity equation is

(5) $$\begin{array}{c} {\dot{n}}_{i}=\frac{1}{e}\;\left(J_{i-1\to i}-J_{i\to i+1}\right),\;i=1,\ldots ,N, \end{array}$$

where the tunneling current density is [7]

(6) $$\begin{array}{ccc} & & J_{i\to i+1}=J_{i\to i+1}^{+}\left(V_{i-1},V_{i},V_{i+1},\mu_{i},T\right)-J_{i\to i+1}^{-}\left(V_{i-1},V_{i},V_{i+1},\mu_{i+1},T\right),\\ & & J_{i\to i+1}^{+}=\frac{e\hbar k_{B}T}{2m_{B}}\sum_{\nu =1}^{n}\int_{0}^{\infty }A_{C1}\left(\epsilon \right)\;A_{C\nu }\;\left(\epsilon +eV_{i}+\frac{ed_{W}\varepsilon_{B}}{4d_{B}\varepsilon_{W}}\left(V_{i-1}+V_{i+1}+2V_{i}\right)\right)\\ & & \;\;\;\;\times \;B_{i-1,i}\left(\epsilon \right)\;B_{i,i+1}\left(\epsilon \right)\;T_{i}\left(\epsilon \right)\;\ln\;\left(1+e^{\frac{\mu_{i}-\epsilon }{k_{B}T}}\right)d\epsilon ,\\ & & J_{i\to i+1}^{-}=\frac{e\hbar k_{B}T}{2m_{B}}\int_{0}^{\infty }\;A_{C1}\left(\epsilon \right)\;A_{C1}\;\left(\epsilon +eV_{i}+\frac{ed_{W}\varepsilon_{B}}{4d_{B}\varepsilon_{W}}\left(V_{i-1}+V_{i+1}+2V_{i}\right)\;\right)\;B_{i-1,i}\left(\epsilon \right)\\ & & \;\times B_{i,i+1}\left(\epsilon \right)T_{i}\left(\epsilon \right)\ln\;\left[1+\exp\;\left(\frac{1}{k_{B}T}\;\left(\mu_{i+1}-\epsilon -eV_{i}-ed_{W}\varepsilon_{B}\frac{V_{i-1}+V_{i+1}+2V_{i}}{4d_{B}\varepsilon_{W}}\right)\;\;\right)\;\right]d\epsilon .\;\; \end{array}$$

In these equations, energies ϵ are measured from the bottom of the *i*th QW, $V_{i}$ and $V_{w_{i}}$, i=1,…,N, are the respective non-negative QB and QW potential drops, and

(7) $$\begin{array}{ccc} & & B_{i-1,i}=k_{i}\;{\left[d_{W}+\left(\frac{1}{\alpha_{i-1}}+\frac{1}{\alpha_{i}}\right)\;\left(\frac{m_{B}}{m_{W}}\sin^{2}\frac{k_{i}d_{W}}{2}+\cos^{2}\frac{k_{i}d_{W}}{2}\right)\;\right]}^{-1}\;,\\ & & \hbar k_{i}=\sqrt{2m_{W}\epsilon },\;\hbar k_{i+1}=\sqrt{2m_{W}e\left(\frac{\epsilon }{e}+V_{i}+\frac{V_{w_{i}}+V_{w_{i+1}}}{2}\right)},\\ & & \hbar \alpha_{i}=\sqrt{2m_{B}e\;\left(V_{B}-\frac{V_{w_{i}}}{2}-\frac{\epsilon }{e}\right)},\;\hbar \alpha_{i-1}=\sqrt{2m_{B}e\left(V_{B}+\frac{V_{w_{i}}}{2}+V_{i-1}-\frac{\epsilon }{e}\right)},\\ & & \hbar \alpha_{i+1}=\sqrt{2m_{B}e\left(V_{B}-\frac{V_{w_{i}}}{2}-V_{i}-V_{w_{i+1}}-\frac{\epsilon }{e}\right)},\\ & & T_{i}\left(\epsilon \right)={\left[\frac{{\left(k_{i}+k_{i+1}\right)}^{2}}{4k_{i}k_{i+1}}+\frac{1}{4}\left(\frac{m_{B}k_{i}}{m_{W}\alpha_{i}}+\frac{m_{W}\alpha_{i}}{m_{B}k_{i}}\right)\;\left(\frac{m_{B}k_{i+1}}{m_{W}\alpha_{i}}+\frac{m_{W}\alpha_{i}}{m_{B}k_{i+1}}\right)\;\sinh^{2}\left(\alpha_{i}d_{B}\right)\right]}^{-1}.\;\; \end{array}$$

Here, $m_{W}$ and $m_{B}$ are the effective masses at QWs and QBs, respectively, and $\hbar B_{i,i+1}/m_{B}$ are the attempt frequencies related to sequential tunneling through the *i*th QB. The electrons are concentrated on a plane located at the end of each QW. The QW wave number $k_{i}$ depends on the electric potential at the center of the *i*th QW, whereas the QB wave number $\alpha_{i}$ depends on the potential $V_{w_{i}}/2$ at the beginning of the *i*th QB [5,7]. The QBs separating the SL from the emitter and collector contacts have potential drops $V_{0}$ and $V_{N}$, respectively. $eV_{B}$ is the QB height for $V_{wi}=V_{i}=0$. In Equation (6), $T_{i}$, given by Equation (7), is the transmission probability through the *i*th barrier separating QWs *i* and i+1 [7].

The current density at emitter and collector contacts can be assumed to adopt phenomenological Ohm laws:

(8) $$\begin{array}{c} J_{0\to 1}=\sigma_{e}\frac{V_{0}}{d_{B_{e}}},\;J_{N\to N+1}=\sigma_{c}\frac{n_{N}}{N_{D_{N}}}\;\frac{V_{N}}{d_{B_{c}}}. \end{array}$$

Here, $\sigma_{j}$ and $d_{B_{j}}$, j=e,c, are the conductivities and effective lengths of the contacts, respectively. $N_{D_{N}}$ is an effective 2D doping density of the collector.

### 2.3. Discrete Poisson Equations

The Poisson equation yields the QB and QW potential drops:

(9) $$\begin{array}{ccc} \varepsilon_{W}\;\frac{V_{w_{i}}}{d_{W}} & = & \varepsilon_{B}\;\frac{V_{i-1}}{d_{B}}+\frac{e}{2}\left(n_{i}-N_{D}\right)\;,\;n_{i}=\sum_{\nu =1}^{n}n_{i}^{\left(\nu \right)}, \end{array}$$

(10) $$\begin{array}{ccc} \varepsilon_{B}\frac{V_{i}}{d_{B}} & = & \varepsilon_{B}\frac{V_{i-1}}{d_{B}}+e\left(n_{i}-N_{D}\right)\;,\;i=1,\ldots ,N, \end{array}$$

where $\varepsilon_{W}$, $\varepsilon_{B}$ and $N_{D}$ are QW and QB static permittivities, and the 2D intentional doping density at the QWs, respectively [5]. These equations produce the relation

(11) $$\begin{array}{c} \frac{\varepsilon_{W}V_{w_{i}}}{\varepsilon_{B}d_{W}}=\frac{V_{i-1}+V_{i}}{2d_{B}},\;i=1,\ldots ,N. \end{array}$$

Then, the *dc* voltage bias condition is

(12) $$\begin{array}{ccc} V_{\mathrm{dc}} & = & \sum_{i=0}^{N}V_{i}+\sum_{i=1}^{N}V_{w_{i}}=\;\left(1+\frac{\varepsilon_{B}d_{W}}{\varepsilon_{W}d_{B}}\right)\;\sum_{i=0}^{N}V_{i}-\frac{\varepsilon_{B}\left(V_{0}+V_{N}\right)\;d_{W}}{2\varepsilon_{W}d_{B}}. \end{array}$$

We differentiate Equation (10) with respect to time, and eliminate $n_{i}=\sum_{\nu =1}^{n}n_{i}^{\left(\nu \right)}$ by using Equation (5). The result is

(13a) $$\begin{array}{ccc} & & \frac{\varepsilon_{B}}{d_{B}}\frac{dV_{i}}{dt}+J_{i\to i+1}=J\left(t\right),\;\;\;\;i=0,1,\ldots ,N. \end{array}$$

where J(t) is the total current density, which is independent of the QW index.

The time-dependent model consists of the 3N+2 Equations (1), (10), (12), and (13a) [the currents are given by Equation (6)], which contain the 3N+2 unknowns $n_{j}$, $\mu_{j}$ (j=1,…,N), $V_{j}$ (j=0,1,…,N), and *J*. Thus, we have a system of equations which, together with appropriate initial conditions, determine our problem completely and self-consistently. For convenience, we again list the minimal set of equations we need to solve in order to completely determine all of the unknowns:

$$\begin{array}{ccc} & & \frac{\varepsilon_{B}}{d_{B}}\frac{dV_{i}}{dt}+J_{i\to i+1}=J\left(t\right),\;\;\;\;i=0,1,\ldots ,N, \end{array}$$

(13b) $$\begin{array}{ccc} & & \varepsilon_{B}\frac{V_{i}}{d_{B}}=\varepsilon_{B}\frac{V_{i-1}}{d_{B}}+e\;\left(n_{i}-N_{D}\right),\;i=1,\ldots ,N, \end{array}$$

(13c) $$\begin{array}{ccc} & & n_{i}=\frac{m_{W}k_{B}T}{\pi \hbar^{2}}\int_{0}^{\infty }A_{C1}\left(\epsilon \right)\;\ln\;\left(1+e^{\left(\mu_{i}-\epsilon \right)/k_{B}T}\right)d\epsilon ,\;i=1,\ldots ,N, \end{array}$$

(13d) $$\begin{array}{ccc} & & V_{\mathrm{dc}}=\left(1+\frac{\varepsilon_{B}d_{W}}{\varepsilon_{W}d_{B}}\right)\sum_{i=0}^{N}V_{i}-\frac{\varepsilon_{B}d_{W}}{2\varepsilon_{W}d_{B}}\left(V_{0}+V_{N}\right), \end{array}$$

together with the constitutive relations given by Equations (6) and (8).

Additional simplifications of electrostatics and the integrals over the energy ϵ lead to simpler versions of Equations (6) and (13) with $F_{i}\approx V_{i}/d_{B}$ [5,54]:

(14a) $$\begin{array}{ccc} & & \varepsilon \;\frac{dF_{i}}{dt}+J_{i\to i+1}=J\left(t\right), \end{array}$$

(14b) $$\begin{array}{ccc} & & J_{i\to i+1}\left(F_{i},n_{i},n_{i+1}\right)=\frac{e}{l}v^{\left(f\right)}\left(F_{i}\right)\left\{n_{i}-\frac{m^{*}k_{B}T}{\pi \hbar^{2}}\ln\;\left[1+e^{-\frac{eF_{i}l}{k_{B}T}}\;\;\left(e^{\frac{\pi \hbar^{2}n_{i+1}}{m^{*}k_{B}T}}-1\right)\;\right]\;\right\}, \end{array}$$

(14c) $$\begin{array}{ccc} & & n_{i}=N_{D}+\frac{\varepsilon }{e}\left(F_{i}-F_{i-1}\right),\;\varepsilon =\frac{d_{B}+d_{W}}{\frac{d_{B}}{\varepsilon_{B}}+\frac{d_{W}}{\varepsilon_{W}}},\;l=d_{B}+d_{W}, \end{array}$$

(14d) $$\begin{array}{ccc} & & v^{\left(f\right)}\left(F_{i}\right)=\sum_{j=1}^{n}\frac{\frac{\hbar^{3}l\left(\gamma_{C1}+\gamma_{C_{j}}\right)}{2m^{*2}}\;T_{i}\left(E_{C1}\right)}{{\left(E_{C1}-E_{C_{j}}+eF_{i}l\right)}^{2}+{\left(\gamma_{C1}+\gamma_{C_{j}}\right)}^{2}},\;m^{*}=\frac{m_{B}d_{B}+m_{W}d_{W}}{d_{B}+d_{W}}, \end{array}$$

(14e) $$\begin{array}{ccc} & & V_{\mathrm{dc}}=l\sum_{i=0}^{N}F_{i}. \end{array}$$

Alternatively, we can obtain a tunneling current density $J_{\mathrm{hom}}\left(Fl\right)$ by numerically simulating the NEGF equations with periodic boundary conditions for identical bias drop Fl over all SSL periods (or modules in the case of a quantum cascade laser), and use

(15) $$\begin{array}{c} J_{i\to i+1}=J_{\mathrm{hom}}\left(F_{i}l\right)\frac{n_{i}-n_{i+1}e^{-\frac{eF_{i}l}{k_{B}T}}}{N_{D}-N_{D}e^{-\frac{eF_{i}l}{k_{B}T}}}, \end{array}$$

instead of Equation (14b) [6,46].

## 3. Domains, Wave Fronts and Excitable Ideal SSL

For a sufficiently large doping density, the current–voltage curve of SSLs display a number of stable branches of stationary solutions, whereas for lower doping density, it has a flat plateau corresponding to SSOC [3]. In both cases, the SSL field profile comprises domains of low and high field values (LFD and HFD) separated by steep DWs with intermediate values of the field. The DWs are the building blocks of multistable stationary branches and of stable SSOC. They can be approximated by solutions of the discrete Equations (13) or (14) for constant current *J* and large *N*.

### 3.1. Domain Walls and Wave Fronts

**Figure 2:**
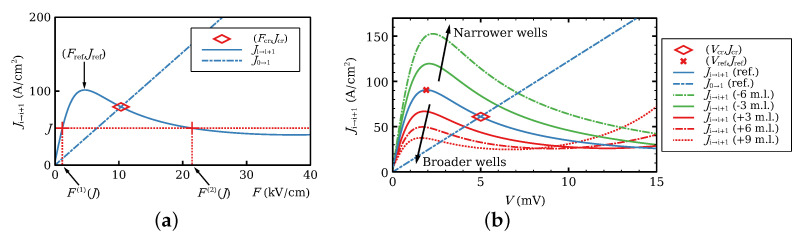
(**a**) Tunneling current density versus field for a homogeneous field $F_{i}=F$ and density $n_{i}=N_{D}$ showing constant solutions $F^{\left(n\right)}\left(J\right)$, n=1,2,3 of $J_{i\to i+1}=J$. (**b**) Tunneling current density versus voltage ($n_{i}=N_{D}$, $V_{i}=V$) comparing the reference configuration (ref.) $d_{B}=4$ nm, $d_{W}=7$ nm to $J_{0\to 1}$ in Equation (8) (dot-dashed straight line) and to configurations having QWs with more or less monolayers (m.l.). The rhombus marks the critical current $J_{\mathrm{cr}}$ and voltage $V_{\mathrm{cr}}$ at which the contact Ohm’s law intersects the reference configuration. When the current surpasses $J_{\mathrm{cr}}$, a new HFD is created at the emitter. Reprinted from E. Mompó, M. Carretero, L. L. Bonilla, Designing hyperchaos and intermittency in semiconductor superlattices, *Physical Review Letters* 127, 096601 (2021); https://doi.org/10.1103/PhysRevLett.127.096601 [28].

Let us consider an infinite SSL at constant current bias *J* and $F^{\left(j\right)}\left(J\right)$, which are as in Figure 2a, i.e., they solve

(16) $$\begin{array}{c} J_{i\to i+1}\left(F,N_{D},N_{D}\right)=J⟹F=F^{\left(n\right)}\left(J\right),\;n=1,2,3. \end{array}$$

**Figure 3:**
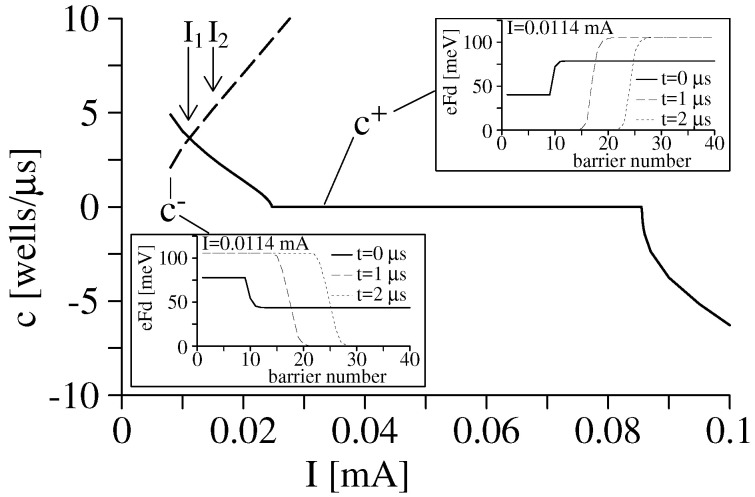
Velocities of wave fronts shown in the inset versus current bias, *I*. GaA/AlAs SSL parameters are $d_{W}=9$ nm, $d_{B}=4$ nm, $N_{D}=1.5\times 10^{11}$ cm^−2^, and cross section $1.13\times 10^{-4}$ cm^2^. Courtesy of Andreas Wacker, appeared in [3].

The discrete Equation (14) have solutions that satisfy $F_{i}\to F^{\left(1\right)}\left(J\right)$ as i→−∞ and $F_{i}\to F^{\left(3\right)}\left(J\right)$ as i→∞ (charge accumulation wave front) or $F_{i}\to F^{\left(3\right)}\left(J\right)$ as i→−∞ and $F_{i}\to F^{\left(1\right)}\left(J\right)$ as i→∞ (charge depletion wave front). These wave fronts (domain walls) satisfy $F_{i}\left(t\right)=F\left(i-ct\right)$, and can be either stationary (pinned wave front with c=0) or moving (depinned wave front with c>0 for smaller values of *J* and c<0 for larger values of *J* past the interval where c=0), as indicated in Figure 3. That there is a finite interval of values of *J*, for which c=0 is a feature of spatially discrete equations, such as (14), whereas the corresponding continuum equations have c=0 for a single value of *J* [27]. The pinning–depinning transition is a continuous global bifurcation that appears in discrete equations [21,25,26,27].

### 3.2. Excitability

**Figure 4:**
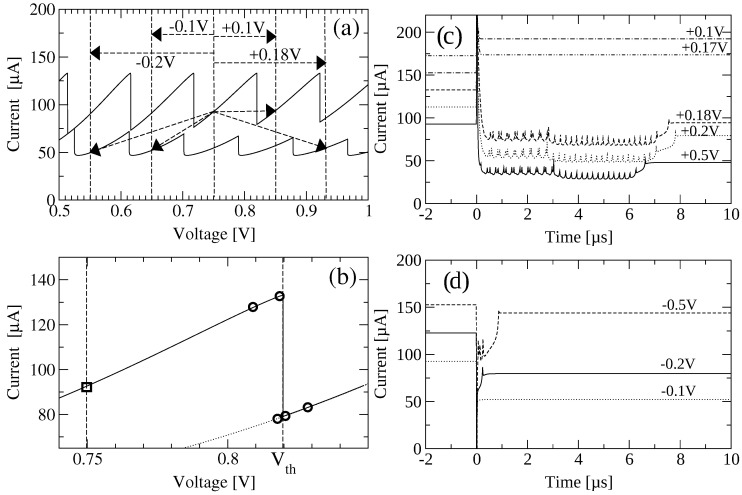
Numerically simulated sawtooth current–voltage characteristic and current response vs. time of a 40-well AlAs/GaAs superlattice ($d_{B}=4$ nm, $d_{W}=9$ nm). Upper branches correspond to voltage up-sweep, lower branches to down-sweep. The arrows in (**a**) indicate the starting and end points of imposed voltage steps. (**b**) gives an enlarged view of the initial operating point $V_{i}=0.75$ V (box), as well as of different final points (circles) below and above the voltage threshold for triggering a large excursion of the current. (**c**) Current vs. time for different initial positive voltage steps. (**d**) Same for negative voltage steps. For clarity, the curves are shifted vertically in units of 20 μA in (**c**) and 30 μA in (**d**). Reprinted from [54].

We now consider the behavior of the SSL under a sudden change in voltage ΔV, as indicated in Figure 4. There are a number of stationary branches that solve Equation (14) with boundary conditions (8). Starting at the voltage marked in Figure 4, we may decrease the voltage toward the values of the preceding branches, or increase it toward larger voltages.

If ΔV<0, the new stable position of the DW separating low and high field domains is to the right of the old one because the LFD is wider, corresponding to a lower voltage. The DW has to move with positive velocity following the flow of the electrons.

**Figure 5:**
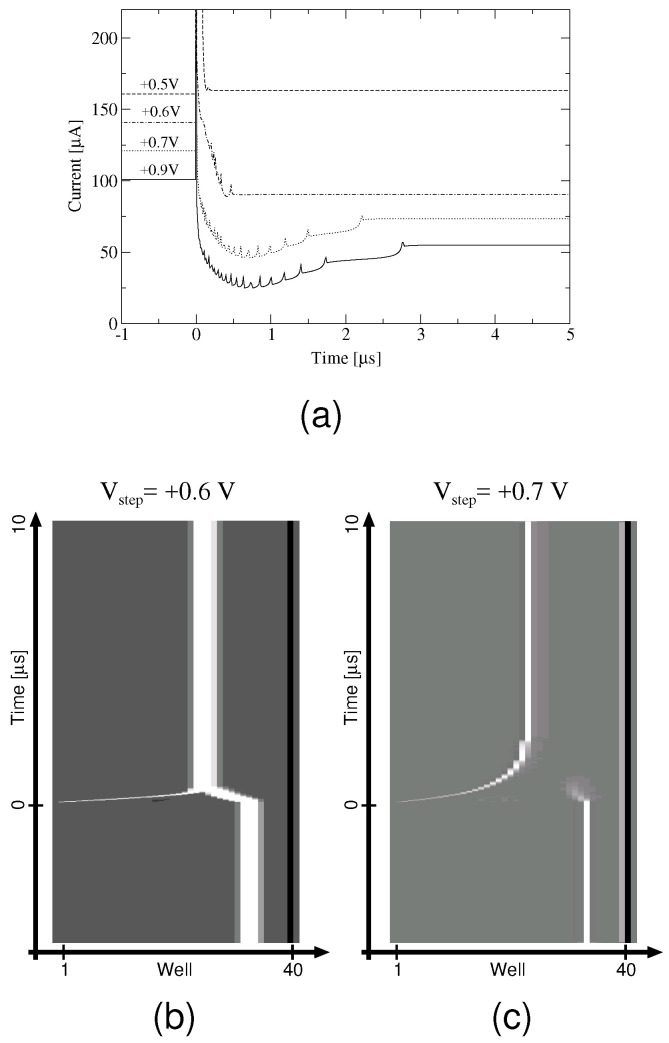
Response of the current (**a**) and evolution of electron densities (**b**,**c**) for different values of ΔV. Reprinted from [54].

If ΔV>0, the SSL response depends on the size of the voltage step. For small ΔV>0, the new stable position of the DW corresponds to the stationary branch just following the old one, and the DW is one SSL period closer to the emitter. To reach this position, the current has to grow to the region of c<0 in Figure 3, the DW (wave front) then moves one SSL period towards the left and stays there. This is shown in Figure 5a,b. If ΔV>0 is so large that the final position of the DW is more than one SSL period towards the emitter, the response is quite different. Firstly, the current increases, and the DW moves one SSL period to the emitter with c<0. Then, a new HFD formed by accumulation and depletion layers (a charge dipole) forms at the emitter, and starts moving towards the collector, while the old DW moves towards the collector, which is a combined charge dipole–monopole (tripole) scenario [54]. To be more precise, we need a quantitative argument [5]. Consider a profile of moving high and low field domains with $n_{+}$ accumulation DWs moving with velocity $c_{+}\left(J\right)>0$ and $n_{-}$ depletion DWs moving with velocity $c_{-}\left(J\right)$. The velocities as functions of the current are shown in Figure 3. If we ignore small regions that contribute little to the voltage, the dc voltage per unit length is

(17) $$\begin{array}{c} \phi =\frac{V_{\mathrm{dc}}}{Nl}=F^{\left(1\right)}\left(J\right)+\left[F^{\left(3\right)}\left(J\right)-F^{\left(1\right)}\left(J\right)\right]\frac{1}{N}\sum_{fronts}\left[X_{-}\left(t\right)-X_{+}\left(t\right)\right], \end{array}$$

where $X_{\pm }\left(t\right)$ are the positions of the DWs. Time differentiation of this equation yields the following evolution equation for the total current density [5]:

(18) $$\begin{array}{ccc} & & \frac{dJ}{dt}=\frac{n_{+}c_{+}\left(J\right)-n_{-}c_{-}\left(J\right)}{N}\;\frac{{\left[F^{\left(3\right)}\left(J\right)-F^{\left(1\right)}\left(J\right)\right]}^{2}}{\frac{F^{\left(3\right)}\left(J\right)-\phi }{v_{1}^{\prime }\left(J\right)}+\frac{\phi -F^{\left(1\right)}\left(J\right)}{v_{3}^{\prime }\left(J\right)}},\;N\gg 1,\\ & & v_{n}^{\prime }\left(J\right)=\frac{\partial }{\partial F}J_{i\to i+1}\left(F^{\left(n\right)}\left(J\right),N_{D},N_{D}\right),\;n=1,2,3. \end{array}$$

These approximate equations do not capture other features seen in numerical simulations and corroborated by experiments [3]. Importantly, each time an accumulation DW crosses a barrier into a new SSL period, the current J(t) displays a small spike. Equation (18) describes a time average that smoothens out these small current spikes.

Just after a large voltage step ΔV>0, the current increases (as shown in Figure 5a) to allow for c<0 in Figure 3. However, when doing that, the current surpasses the critical value (diamond in Figure 2), and a new HFD appears at the emitter. It is formed by an accumulation DW at its back and a depletion DW at its front, moving with velocities $c_{+}\left(J\right)$ and $c_{-}\left(J\right)$, respectively. The old DW moves with velocity $c_{+}\left(J\right)$ towards the collector. Thus, we have $n_{+}=2$, $n_{-}=1$ in Equation (18). The current *J* tends towards the value that solves $2c_{+}\left(J\right)=c_{-}\left(J\right)$, while numerical simulations show double spikes corresponding to the motion of the two DWs. After the old DW reaches the collector, $n_{+}=n_{-}=1$ and the current tends towards the intersection between $c_{+}$ and $c_{-}$ in Figure 3. Simulations show single spikes corresponding to the motion of a single DW. The depletion DW at the front of the dipole continues moving until it exits at the collector. This is shown in Figure 5a,c.

The behavior of the SSL we have described is typical of an excitable medium. What happens if the voltage step is not applied abruptly? If the voltage step ΔV is applied during a certain time Δt, for example by the linear function $\Delta V\left(t\right)=\frac{\Delta V}{\Delta t}t$ for 0<t<Δt, then different scenarios can occur, from adiabatic switching from one stationary branch to another, to triggering of tripole scenarios and skipping stationary branches; see [55].

## 4. Self-Sustained Oscillations of the Current

**Figure 6:**
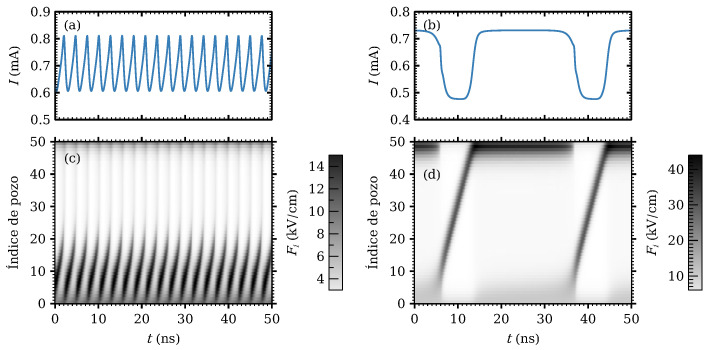
Evolution of the current (**a**,**b**) and of the electric field profile (**c**,**d**) during typical high- and low-frequency SSOCs on the left and right panels, respectively. High frequency oscillations correspond to a supercritical Hopf bifurcation (left panel), whereas low frequency oscillations appear near the SNIPER bifurcation (right panel). Reprinted from the supplementary material of [29].

Equation (18) is also the key to understanding the oscillatory behavior of SSLs. Depending on the composition, doping density, temperature, and configuration of the SSL, it may behave as an excitable medium, as explained in the previous section, or it may exhibit stable oscillations of the current under *dc* voltage bias [3]. In the latter case, under *dc* voltage bias and for a sufficiently large *N* (N≥14 for the SSL of Figure 6), the oscillations occur on an intermediate range of voltages, outside which the SSL is in a stable stationary state. As $V_{\mathrm{dc}}$ increases, the SSOC appear as a supercritical Hopf bifurcation and finish at either a Hopf or a SNIPER bifurcation. The corresponding field profiles are repeated generation of HFDs at the emitter that either die before reaching the collector (lower part of the SSOC voltage interval) and have high frequency, or reach the collector and have low frequency; see Figure 6. A SNIPER bifurcation is observed in experiments [56] and in numerical simulations [29].

For larger voltages, a typical stable oscillation of the current in a long weakly coupled SSL is a relaxation oscillation with periods having different stages. In the first stage, a HFD bounded by accumulation and depletion DWs moves towards the collector. According to Equation (18), the current evolves rapidly to the value $J^{*}$ where $c_{+}\left(J\right)=c_{-}\left(J\right)$ in Figure 3. When the depletion DW reaches the collector, $n_{+}=1,n_{-}=0$, and J(t) increases according to Equation (18). When the current surpasses the critical value in Figure 3, a new HFD is triggered at the emitter. Then, $n_{+}=2,n_{-}=1$, and the current decreases to the value such that $2c_{+}\left(J\right)=c_{-}\left(J\right)$. When the accumulation layer of the original HFD reaches the collector, $n_{+}=n_{-}=1$ and we are back at the first stage, having completed a period of the oscillation. These stages are illustrated in Figure 6 that shows the evolution of the current and of the electric field profiles during one oscillation period. What happens for large voltages close to the SNIPER bifurcation is that the HFD close to the collector never exits and the new HFD triggered when the current increases past its critical value has a shorter motion between the emitter and the accumulation DW of the exiting HFD as shown in Figure 6. For voltages past the SNIPER bifurcation, the current falls below the critical value (the saddle-node part of the SNIPER), new HFD are not triggered at the emitter and only a stable stationary LFD followed by a HFD next to the collector remains.

Depending on the emitter conductivity, the previous scenario can be modified. If the conductivity is very large, the current $J_{0\to 1}$ does not intersect $J_{i\to i+1}\left(F,N_{D},N_{D}\right)$. Then, SSOCs are due to the dynamics of accumulation DWs, according to numerical simulations [3,5]. If the emitter conductivity is fine-tuned, it is possible to trigger several HFD and the oscillations become more complicated, even chaotic [3,57].

## 5. Effects of Noise: Coherence and Stochastic Resonances

So far, we have not considered the very important effects of internal and external noise and disorder on the nonlinear phenomena in SSLs. Here, we consider the effects on an ideal SSL of an external noise provided by a voltage source. Internal shot and thermal noises produce much weaker effects, and can be ignored. Thus, the SSL can be described by Equation (14) with

(19) $$\begin{array}{c} l\sum_{i=0}^{N}F_{i}=V+\eta \left(t\right),\;\eta \left(t\right)=\eta_{\mathrm{th}}\left(t\right)+\eta_{c}\left(t\right), \end{array}$$

replacing Equation (14e), or by Equation (13) with

(20) $$\begin{array}{c} \left(1+\frac{\varepsilon_{B}d_{W}}{\varepsilon_{W}d_{B}}\right)\sum_{i=0}^{N}V_{i}-\frac{\varepsilon_{B}d_{W}}{2\varepsilon_{W}d_{B}}\left(V_{0}+V_{N}\right)=V+\eta \left(t\right),\;\eta \left(t\right)=\eta_{\mathrm{th}}\left(t\right)+\eta_{c}\left(t\right), \end{array}$$

replacing Equation (13d). In these equations, *V* may consist of the *dc* voltage bias $V_{\mathrm{dc}}$ and an *ac* signal $V_{\mathrm{ac}}=V_{\sin}\sin\left(2\pi \nu t\right)$ ($V_{\sin}=\sqrt{2}\;V_{\sin}^{\mathrm{rms}}$), whereas η(t) has one component, $\eta_{\mathrm{th}}\left(t\right)$, related to the noise of the source, and the external noise $\eta_{c}\left(t\right)$. $\eta_{\mathrm{th}}\left(t\right)$ is simulated by picking random numbers every $5\times 10^{-11}$ s from a zero mean distribution with a standard deviation of 2 mV. $\eta_{c}\left(t\right)$ is a white noise with bandwidth of 1 GHz and tunable amplitude $V_{\mathrm{noise}}^{\mathrm{rms}}$ [29]. For these noise values, numerical simulations of the model agree qualitatively with the results of the experiments reported in [56]. In the present paper, we have simulated numerically the detailed Equation (13) (corresponding to having different effective mass and permittivities at barriers and wells) with the voltage bias of Equation (20). In our previous work [29], we solved Equations (14) and (19) for average mass and permittivities. The results of numerical simulations of both detailed and averaged rate equations are qualitatively the same and quantitatively quite similar. Compared to experiments, it is known that the total current and the frequency of its SSOC are lower than observed [3].

We study an ideal AlGaAs/GaAs 50-period SSL at room temperature with three populated subband energies, 41.6, 165.8, and 354.3 meV, calculated using Equation (4). The subband broadenings due to scattering are 2.5, 8, and 24 meV, respectively, and the 2D doping density is $N_{D}=6\times 10^{10}$ cm^−2^ [55]. The SSL has cross section $A=s^{2}$ with s=30 μm, and $m_{W}=0.063\;m_{e}$, $m_{B}=\left(0.063+0.083x\right)m_{e}=0.1m_{e}$ (for x=0.45), $d_{B}=4$ nm, $d_{W}=7$ nm, $l=d_{B}+d_{W}$, $\varepsilon_{B}=10.9\epsilon_{0}$, $\varepsilon_{W}=\left(12.9-2.84x\right)\epsilon_{0}$, $\epsilon_{0}$, and $V_{\mathrm{dc}}$ are the effective electron mass at QW and QBs, the (Al,Ga)As QB thickness, the GaAs QW thickness, the SSL period, the QB permittivity, the QW permittivity, the dielectric constant of the vacuum, and the *dc* voltage bias, respectively. We select contact conductivities $\sigma_{c}=\sigma_{e}=0.49$ A/Vm and the same doping density $N_{D}$ for emitter and collector.

**Figure 7:**
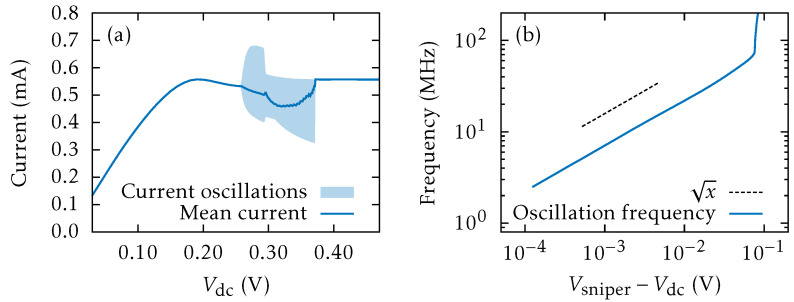
(**a**) Current–voltage diagram of the numerically simulated AlGaAs/GaAs SSL. Maximum, minimum and time-averaged values of the current are shown for voltages on the interval of SSOC. (**b**) Frequency of the SSOC near the voltage $V_{\mathrm{SNIPER}}$ that shows the square root dependence characterizing a SNIPER bifurcation.

Figure 7a shows the current versus *dc* voltage diagram. For η(t)=0, the branch of SSOC appears as a supercritical Hopf bifurcation at $V_{\mathrm{dc}}=0.257$ V and ends at a SNIPER at $V_{\mathrm{dc}}=0.372$ V. These values are lower than observed experimentally, as we have not included the large resistor in series with the SSL sample and more realistic contact boundary conditions [56]. Near the SNIPER, the oscillation frequency is proportional to $\sqrt{V_{\mathrm{SNIPER}}-V_{\mathrm{dc}}}$, as shown in Figure 7b. For larger voltages, there remains a stable stationary profile comprising a LFD followed by a HFD that ends at the collector. This is the same scenario of the experiments [56]. Earlier experiments had demonstrated CR on a AlAs/GaAs SSL at 77K by adding a white noise $\eta_{c}\left(t\right)$ of varying strength in Equation (19) [58].

In nonlinear excitable systems [12], noise of appropriate strength can trigger coherent oscillations, a CR, and enhance the signal-to-noise ratio of a periodically driven bistable system, an SR. These effects of noise are typically demonstrated in systems of few degrees of freedom that allow for analytical and simple numerical studies. A particle in a double-well potential under white noise and *ac* driving forces experiences SR [59], whereas a CR is demonstrated in the excitable FitzHugh–Nagumo equation [60,61]. For an AlAs/GaAs SSL at ultralow temperature, which is a system with many degrees of freedom, Hizanidis et al. predicted CR by numerically simulating Equation (14) with added internal noise of tunable intensity [62]. They placed the voltage close to a SNIPER bifurcation on the side where a stationary state is stable. Then, they increased the strength of their internal noise and demonstrated numerically CR.

**Figure 8:**
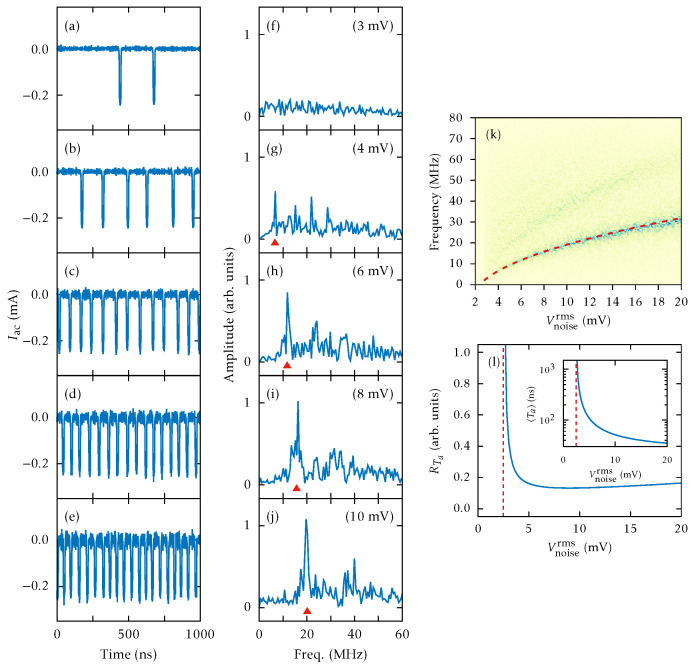
Coherence resonance: (**a**–**e**) *ac* components of the time dependent current, and (**f**–**j**) the corresponding frequency spectra (the triangle marks the interspike average frequency) for different noise amplitudes at $V_{\mathrm{dc}}=0.373$ V. Values of $V_{\mathrm{noise}}^{\mathrm{rms}}$ are 3, 4, 6, 8, and 10 mV. Current traces have been shifted to have zero current at the stationary state. (**k**) Fourier spectra of the current traces J(t) for different values of the bandlimited white noise RMS amplitude. Darker (brighter) colors represent higher (lower) frequency amplitudes (in arbitrary units). The frequency associated with the mean interspike time is represented by a dashed red line. (**l**) Normalized standard deviation versus $V_{\mathrm{noise}}^{\mathrm{rms}}$ noise ($\eta_{\mathrm{th}}=0$). Inset: mean interspike interval versus $V_{\mathrm{noise}}^{\mathrm{rms}}$. The vertical asymptotes (dashed lines) occur at $V_{\mathrm{noise}}^{\mathrm{rms}}=2.494$ mV.

When we impose the same external noise (20) as in the experiments [56], CR arises past the SNIPER at $V_{\mathrm{dc}}=0.372$ V. Adding noise with increasing amplitude at $V_{\mathrm{dc}}=0.373$ V, a CR appears as coherent SSOC; see Figure 8a–e for $V_{\mathrm{noise}}^{\mathrm{rms}}\geq 4$ mV. For smaller values, as shown in Figure 8a, the noise randomly triggers current spikes corresponding to the emission of an HFD (charge dipole wave) at the injecting contact and its motion towards the collector. However, the frequency spectrum does not exhibit an appreciable peak; see Figure 8f. For larger values of $V_{\mathrm{noise}}^{\mathrm{rms}}$, larger current spikes form a periodic pattern and a large peak appears in the frequency spectrum, which indicates a CR. Its frequency follows the interspike average frequency [marked by triangles in Figure 8f–j] produces the CR frequency, which increases with $V_{\mathrm{noise}}^{\mathrm{rms}}$.

Figure 8k shows the Fourier spectra of J(t) for different values of $V_{\mathrm{noise}}^{\mathrm{rms}}$. The dashed red line indicates the frequency associated with the mean interspike time. For $V_{\mathrm{noise}}^{\mathrm{rms}}\leq 3$ mV, the noise causes a rapid small-amplitude oscillation of the current and large spikes separated by long-time intervals. Between spikes, the current is close to a constant value slightly above the critical current defined in Figure 2. Each large spike of the current corresponds to triggering a HFD (charge dipole) that moves towards the collector, as explained in the excitability scenario of Section 3. The field profile for currents different from large spikes is quasistationary: $F_{i}\approx F_{\mathrm{cr}}$ near the emitter, then $F_{i}$ decreases to $F^{\left(1\right)}\left(J\right)$, stays there for several periods, and increases again near the collector.

Figure 8l depicts the normalized standard deviation $R_{T_{a}}=\sqrt{\langle T_{a}^{2}\rangle -{\langle T_{a}\rangle }^{2}}/\langle T_{a}\rangle$ of the interspike time interval, $T_{a}$, versus $V_{\mathrm{noise}}^{\mathrm{rms}}$. It has a minimum after an abrupt drop, followed by a smooth increase. This behavior is expected for voltages close to a SNIPER bifurcation [62]. The inset of Figure 8l shows that the mean interspike interval $\langle T_{a}\rangle$ first decreases from infinity at $V_{\mathrm{noise}}^{\mathrm{rms}}=2.294$ mV and rather more smoothly for $V_{\mathrm{noise}}^{\mathrm{rms}}>4$ mV. This behavior agrees qualitatively with the experimental results [56].

**Figure 9:**
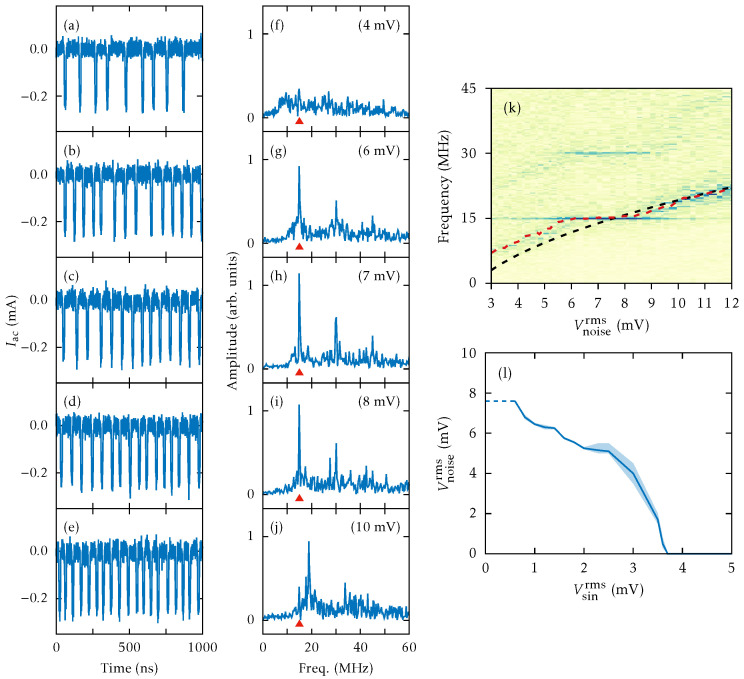
Stochastic resonance: (**a**–**j**) are as in Figure 8, but now a 15 MHz *ac* signal with $V_{\sin}^{\mathrm{rms}}=1.8$ mV has been added. The values of $V_{\mathrm{noise}}^{\mathrm{rms}}$ are 4, 6, 7, 8, and 10 mV. (**k**) Fourier spectra of J(t) for different values of $V_{\mathrm{noise}}^{\mathrm{rms}}$. Darker (brighter) colors represent higher (lower) frequency amplitudes (in arbitrary units). The frequency associated with the mean interspike time is represented by a dashed red line and the CR frequency is indicated by a dashed black line. (**l**) Values of $V_{\mathrm{noise}}^{\mathrm{rms}}$ needed to trigger periodic SSOC versus $V_{\sin}^{\mathrm{rms}}$.

Having shown the existence of a CR, we can add a weak sinusoidal signal to the bias and try to see whether it resonates with the CR for appropriate noise, giving rise to a stochastic resonance, and can be discovered. This is shown in Figure 9a–e: a small amplitude *ac* signal with a frequency within the CR range is added to the *dc* voltage and the noise amplitude is increased. For $V_{\mathrm{noise}}^{\mathrm{rms}}<4$ mV, there appear large current spikes separated irregularly by long intervals. Figure 9g–i show that the SSL oscillates at a frequency locked with that of the *ac* signal, as the noise amplitude increases. At larger $V_{\mathrm{noise}}^{\mathrm{rms}}$, Figure 9j shows that the main frequency increases and ceases to be locked to that of the *ac* signal. This is the signature of a SR, which is also observed in experiments [56].

Figure 9k shows the Fourier spectra of the current traces J(t), including that of the 15 MHz sinusoidal *ac* signal. We observe how the frequency associated with the mean interspike time and the CR frequency come together as $V_{\mathrm{noise}}^{\mathrm{rms}}$ increases. Figure 9l shows the values of $V_{\mathrm{noise}}^{\mathrm{rms}}$ that trigger periodic SSOC versus the amplitude of the sinusoidal signal, $V_{\sin}^{\mathrm{rms}}$. Clearly, $V_{\mathrm{noise}}^{\mathrm{rms}}$ decreases as the amplitude of the *ac* signal $V_{\sin}^{\mathrm{rms}}$ increases, a trend that is also observed in experiments [56]. As explained in the latter reference, the SR can produce an enhancement of the signal-to-noise ratio by more than 30 dB [56]. Thus, excitable SSL devices could be used to amplify weak *ac* signals, similarly to lock-in amplifiers that amplify and detect weak *ac* signals immersed in strong background noise.

## 6. Modified Superlattices and Robust Chaos

Chaotic SSOC under *dc* [63] or *dc + ac* [64] voltage bias were first observed at ultralow temperatures in GaAs/AlAs SSL. Theoretical predictions using spatially discrete models of ideal SSL anticipated experimental observations in the *ac*-driven case [65], but not for spontaneous chaos. The latter was associated with fine tuning of the conductivity of the emitter in ideal weakly coupled SSLs (so that a variable number of HFDs can be triggered at the emitter) [57], or to transitions between different oscillation modes at thin intervals of *dc* voltage (HFDs that die before arriving to the collector versus HFDs that arrive at the collector before a new HFD is triggered) [66]. In the latter case, chaos can be enhanced or induced by noise [66,67].

A change in the composition of QBs has resulted in the observation of SSOC at room temperature in GaAs/Al_0.45_Ga_0.55_As SSLs [8]. SSOCs include time periodic, quasiperiodic and chaotic oscillations, but the latter occur for wider voltage intervals than those predicted by numerically simulating ideal SSLs with identical periods [8]. This could be due to disorder related to growth techniques, because it is empirically known that the ability to display chaos varies from sample to sample. In this section, we explain how to exploit this observation to enhance chaos by appropriate design of the SSL [7,28].

Why is chaos important? Quantum tunneling of electrons is a random unpredictable process at the microscopic level [68]. This essential randomness is enhanced by collective transport of the electrons at the macroscopic level. Then, the entropy generated by tunneling is enhanced by collective transport and it can produce true random sequences, not pseudorandom sequences as produced, e.g., by computers. Thus, chaos in SSLs can be used to build fast generators of true random sequences, which can be used in safe data transmission and storage, encryption, electronic commerce [69,70,71,72], stochastic simulation [73], Monte Carlo simulations [74], etc.

How can we enlarge the voltage intervals where the SSL response is chaotic? On ideal SSLs, chaos is associated with the transition between high-frequency small-amplitude current oscillations corresponding to HFDs that die before arriving at the collector and low-frequency large-amplitude oscillations corresponding to HFDs arriving at the collector and being recreated at the emitter [66]. The voltage window for this type of chaos is necessarily narrow. The mechanism of triggering many HFDs by modifying the emitter conductivity [57] is not feasible as it is not possible to control the contact conductivity with the necessary accuracy. Thus, our main idea is to change the SSL so that HFDs (charge dipole waves) can be triggered at different QWs of the sample. To this end, it is important that we understand how to generate chaotic attractors on wide voltage intervals by appropriate design [7,28]. This topic has been discussed extensively in [7], to which we refer for details. We give a shortened discussion below adapted from [7].

### 6.1. Inserting Wider Wells on the Ideal SSL

Figure 2b shows the current−voltage curve for the reference configuration of an ideal SSL (with $n_{i}=N_{D}$, voltage *V*, $d_{B}=4$ nm, $d_{W}=7$ nm), the resulting curves when we add or subtract a number of monolayers (0.3 nm wide each) to $d_{W}$, and Ohm’s law at the contact, $J_{0\to 1}\left(V\right)$. $J_{i\to i+1}\left(V\right)$ exhibits a single maximum at the shown voltage range. Widening (respectively, narrowing) the well decreases (respectively, increases) the maximum and shifts it toward lower (respectively, higher) voltages. The intersection of $J_{i\to i+1}\left(V\right)$ and $J_{0\to 1}\left(V\right)$ (marked with a rhombus for the reference configuration) changes accordingly. This intersection roughly marks the voltage and current at which the contact issues a dipole wave (HFD) [3,5,6,7,28]. We surmise that lowering the threshold to trigger HFDs should enrich their dynamics, which implies inserting wider QWs with more than six extra monolayers [7]. As explained in [7], it is convenient to insert modified QWs having 10 extra monolayers (i.e., $d_{W}=10$ nm). The first three energy levels (subbands), given by solving Equation (4), are $E_{C_{1}}=24.0$ meV, $E_{C_{2}}=96.1$ meV, $E_{C_{3}}=214.7$ meV.

If we put a single modified QW at $i=i_{w}$, there appear SSOCs for sufficient voltage. They are repeatedly nucleated at $i_{w}$, move toward the collector and disappear there, provided the voltage is large enough and the number of SSL periods surpasses the minimum number of SSL periods required for oscillations [3,5]. Numerical simulations show that the SSOC are similar to those of the unmodified ideal SSL, except that the charge dipoles move over a smaller part of the SSL. No chaos is observed [7].

Suppose we insert two modified QWs at $i_{1}$ and $i_{2}$ ($i_{1}<i_{2}<N$), with $d_{W_{j}}=10$ nm, j=1,2. If the wider wells are different, the resulting dynamics is similar to that of one modified well, for one of the modified wells will dominate the other [7]. If both QWs are identical, we denote the intervals $i<i_{1}$, $i_{1}<i<i_{2}$, and $i>i_{2}$ as regions I, II, and III, respectively. We fix $i_{1}=5$, so that dipole nucleation occurs at $i_{1}$ and not at the injector [7]. We then vary $i_{2}$. If regions II and III have more than 14 wells, dipole waves can be nucleated at $i_{1}$ and at $i_{2}$, they travel through regions II and III, respectively, and their motion is strongly correlated. In general, each region II and III can support only one dipole wave. By tuning $i_{2}$, we have observed different types of chaos in numerical simulations. Here, we give a general idea of the results while a detailed study can be found in [7].

**Figure 10:**
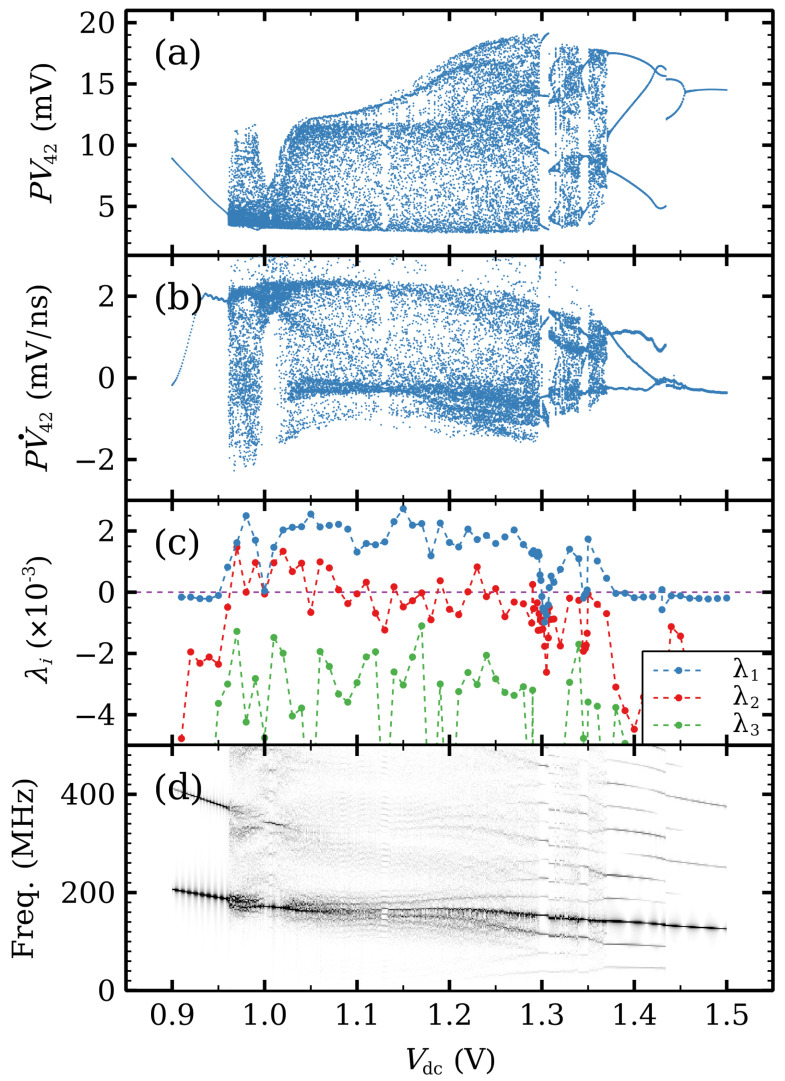
Poincaré maps from (**a**) $V_{42}\left(t\right)$ and (**b**) ${\dot{V}}_{42}\left(t\right)$, (**c**) Lyapunov exponents, and (**d**) Fourier spectrum as functions of $V_{\mathrm{dc}}$ for the modified SSL with $i_{1}=5$ and $i_{2}=30$. There are jumps between periodic attractors at $V_{dc}=1.3\;V$ and $V_{dc}=1.43\;V$ (Poincaré map) corresponding to quasi-periodic attractors with different incommensurate frequencies (Fourier spectrum). There is hyperchaos (2 positive Lyapunov exponents of comparable magnitude) for $V_{dc}<1.08\;V$ and intermittent chaos for $V_{dc}>1.08\;V$ ($\lambda_{1}\gg \lambda_{2}\approx 0$). Reprinted from E. Mompó, M. Carretero, L. L. Bonilla, Designing hyperchaos and intermittency in semiconductor superlattices, *Physical Review Letters* 127, 096601 (2021); https://doi.org/10.1103/PhysRevLett.127.096601 [28].

Figure 10 shows the rich dynamical behavior on the SSOC voltage range. Taking the time traces of two well-separated periods, Figure 10a,b show the Poincaré maps constructed by plotting $V_{42}\left(t\right)$ and of ${\dot{V}}_{42}\left(t\right)$, respectively, at times $t^{*}$, where $V_{12}\left(t\right)$ takes on its mean value in time and ${\dot{V}}_{12}\left(t^{*}\right)>0$. In contrast to spontaneous chaos in shorter ideal SSLs, chaotic oscillations do not ensue through the Feigenbaum period-doubling cascade scenario [75]. Instead, chaos is related to quasi-periodicity. Firstly, a supercritical Hopf bifurcation yields a periodic attractor, whose field profile exhibits repeated nucleation of HFDs at the $i_{1}$ and $i_{2}$ QWs that die before reaching the end of their respective regions [7]. The resulting high frequency is displayed in the density plot of the normalized Fourier spectrum of Figure 10d. At $V_{\mathrm{dc}}=0.96$ V, another periodic attractor appears and interacts with the first one, resulting in a hyperchaotic attractor with two positive Lyapunov exponents, as shown in Figure 10c. In the hyperchaos interval $0.961<V_{\mathrm{dc}}<1.1$, HFDs nucleated at the $i_{2}$ QW either do not reach the collector or, if they do, HFDs cannot stay in the QWs near the collector [7]. For $1.10<V_{\mathrm{dc}}<1.37$, there appears intermittent chaos corresponding to the emergence of another cycle that interacts with the others and eventually disappears at a saddle point [7]. The intermittency comprises irregular bursts corresponding to a cycle separated by intervals for which the trajectories are close to the saddle point. The quiescent stage of intermittency is associated with HFDs that reach the collector, stop there and remain in the last SSL periods. The periodic bursts are associated with recycling of HFDs in Regions II and III. On the intermittency interval, the second Lyapunov exponent is smaller, but still positive. At $V_{\mathrm{dc}}=1.2$ V, the saddle point expands to a saddle cycle and the intermittent behavior now has low frequency oscillations at the quiescent stage [7]. At $V_{\mathrm{dc}}=1.37$ V, the intermittency becomes a period 3 cycle (three loop trajectories in the phase plane). At larger *dc* voltages, chaos has disappeared and the periodic behavior becomes even simpler (two loops at 1.43 V, a single loop for larger voltages). The transition from periodic attractors with three loops to two loop ones at 1.43 V is rather abrupt, as shown in Figure 10a,b. Sweeping up or down the *dc* voltage about 1.43 V, we have detected a hysteresis cycle about this value. The time periodic SSOC disappear at a supercritical Hopf bifurcation.

### 6.2. Effects of Disorder and Noise on Chaotic Attractors

To build feasible devices with chaotic behavior due to insertion of two modified QWs, we need to discuss how noise and disorder change SSL SSOC. Let us consider disorder first. When growing SSLs, it is difficult to control perfectly the width of the layers of the two semiconductors. To account for unintended growth disorder, we add random numbers to QW widths, selecting them from a zero mean normal distribution with standard deviation σ, and numerically solve the SSL model. Depending on the resulting disordered configuration, intervals of hyperchaos or intermittent chaos are either destroyed or remain. When there are long voltage intervals where the chaotic behavior of the SSL without disorder remains, we consider these examples as successes. If disorder changes chaos in ideal SSLs to time periodic or stationary solutions, we consider these examples as failures. For a given σ, the success rate of disordered SSLs that still exhibit chaotic behavior is shown in Figure 10 of [7]. For σ<0.015 nm, chaotic attractors observed for the SSL without disorder remain. However, σ=0.024 nm is sufficient to have a lower success rate of 70%.

To design SSL devices with voltage windows of chaos, we need to control disorder effects as well as possible. During epitaxial growth [2], Al atoms within each interface alloy monolayer may be segregated into local clusters or not be randomly positioned in the Ga or the As sublattice [76]. This yields a nonzero σ, even if there are no errors in the number of monolayers per QB and QW (recall that the monolayer width is 0.3 nm). It is possible to attain σ<0.018 nm in simpler devices [76,77], which would yield reliably chaotic SSLs according to the success rate of Figure 10 in [7].

In sharp contrast with chaos in ideal SSLs [66], the Lyapunov exponents of modified SSLs are not affected by internal noise (shot and thermal noise) and external voltage noise (2 mV rms for a 50 Ohm resistor) [7]. For hyperchaos, noise does not change the two largest Lyapunov exponents, but it produces a dispersion near their deterministic values, with larger standard deviation for the second largest exponent. For intermittent chaos, the two largest Lyapunov exponents are noticeably smaller than their values in the absence of noise. The explanation could be that noise forces the system to visit more often contraction regions of the phase space, such as the quiescent regions between bursts in intermittent chaos, which lowers the largest Lyapunov exponent [78]. The third Lyapunov exponent may increase with noise, but it remains negative [7].

## 7. Conclusions

In weakly coupled SSLs, the main transport mechanism is resonant tunneling. Here, we have reviewed a detailed theory of nonlinear transport based on a hierarchy of time scales: from the shortest intrasubband relaxation times to intersubband scattering times, to the much larger dielectric relaxation times [33]. Time scale separation justifies describing SSL behavior by the spatially discrete rate Equation (13) for voltage drops at QBs and QWs and for electron densities [7]. These equations can be further simplified to Equation (14) for the average electric fields and electron densities at QWs [5]. Theories based on density matrices or NEGFs yield different expressions for the tunneling current [6], but they still assume the same hierarchy of time scales. A first-principles derivation of spatially discrete transport equations does not exist at the present time.

Using Equation (14), we show that weakly coupled SSLs are excitable media [3,54]. For large doping density, the I−V curve displays a number of branches corresponding to stable stationary states. Applying an abrupt voltage step, the current of the SSL either goes rapidly to a stationary state or it performs a large excursion before reaching the latter. This excursion corresponds to the generation of a HFD (charge dipole wave) at the emitter contact and its motion to the collector [54]; see [55] for the effects of applying a time dependent voltage step.

Noise in some excitable systems is known to produce a stable oscillatory state when its strength is appropriate, which is a coherence resonance [60,61]. In SSLs, coherence resonance was predicted in [29] and observed experimentally in [56]. The prediction was the result of numerically simulating Equations (14) and (19), whereas here, we have studied the same phenomenon using the detailed Equations (13) and (20). The results are qualitatively the same, although we require larger noise strengths in the more detailed model used here to obtain CR. These values are closer to (albeit still smaller than) those found in the experiments [56]. Once we have found that noise produces CR, it is natural to see whether we can use noise to detect weak signals by stochastic resonance with it [59]. SR is predicted by the numerical simulations of the detailed model we use here, by the averaged model [29], and it has been experimentally demonstrated at room temperature in [56]. Thus, these excitable SSL devices could be used to amplify and detect weak *ac* signals immersed in strong background noise.

Lastly, we have reviewed our previous results of attaining robust chaotic dynamics by insertion of two identical and wider QWs in appropriate periods of a SSL [7]. Chaos in SSL can be used to produce fast sequences of true random numbers [8,69], which are useful for safe storage and transmission of data [71,72], encryption via chaos synchronization [79,80], etc. In the modified design of the SSL, hyperchaos and chaos by intermittencies are the result of triggering dipole waves at the modified QWs and their interaction. Adding noise and disorder within bounds achievable with current technology does not change chaotic dynamics [7,28]. Thus, our design of robust chaos could be used to obtain more reliable chaotic dynamics in SSLs. It remains to explore whether insertion of more identical wider QWs in longer SSLs increases the number of Lyapunov exponents of chaotic attractors, and whether such SSLs could be successfully grown.

## Data Availability

The original contributions presented in the study are included in the article, further inquiries can be directed to the corresponding author.

## Abbreviations

The following abbreviations are used in this manuscript:

1D, 2D	One dimensional, two dimensional
CR	Coherence resonance
DW	Domain wall
HFD	High field domain
LFD	Low field domain
QB	Quantum barrier
QW	Quantum well
SNIPER	Saddle-node infinite period
SSOC	Self-sustained oscillations of the current
SSL	Semiconductor superlattice
SR	Stochastic resonance
